# Cessation of Exclusive Breastfeeding and Determining Factors at the University of Gondar Comprehensive Specialized Hospital, Northwest Ethiopia

**DOI:** 10.1155/2020/8431953

**Published:** 2020-03-20

**Authors:** Bayew Kelkay, Eshetie Kindalem, Animut Tagele, Yohannes Moges

**Affiliations:** ^1^School of Midwifery, College of Medicine and Health Sciences, University of Gondar, Gondar, Ethiopia; ^2^Department of Midwifery, Institute of Medicine and Health Sciences, DebreBerhan University, DebreBerhan, Ethiopia

## Abstract

**Background:**

Exclusive breastfeeding (EBF) is the gold standard of infant feeding practice which lasts up to 6 months postpartum. Not all infants are exclusively breastfed in developing countries, including Ethiopia. This study, therefore, assessed the magnitude and determining factors of EBF cessation practice among mothers at University of Gondar Comprehensive Specialized Hospital, Northwest Ethiopia.

**Method:**

Institutional-based cross-sectional study design using a systematic random sampling technique was applied to select 344 mothers of infants aged 9 months came for measles vaccination. Pretested structured questionnaire was used to collect the data. Data were entered, cleaned, and analyzed by using SPSS version 21. Independent variables with a *P* value of <0.05 indicated association.

**Result:**

The magnitude of cessation of EBF was 21.5% with 95% CI (17.24-25.76). Maternal age ≤ 19 years [AOR = 5.53; 95% CI (1.07-28.57)], civil servants [AOR = 4.73; 95% CI (2.20-10.19)], illiterate husbands [AOR = 3.76; 95% CI (1.13-12.49)], primi-para [AOR = 2.42; 95% CI (1.22-4.79)], no postnatal follow up [AOR = 2.62; 95% CI (1.44-4.80)], and having poor knowledge on breastfeeding benefits and composition of breastmilk [AOR = 3.15; 95% CI (1.56-6.35)] were independent factors significantly associated with cessation of EBF. *Conclusion and recommendation*. The magnitude of cessation of EBF was high. Maternal age, parity, employment status, postnatal follow-up, and breastfeeding knowledge as well as spouse literacy level were independent factors significantly associated with cessation of EBF. Our study provides further impetus for empowering young and primi-para with breastfeeding knowledge, an extension of maternity leave time, and support for breastfeeding at the workplace.

## 1. Introduction

Breast milk is natural and renewable food that is environmentally sound, produced, and delivered to consumers without pollution, unnecessary packaging, or waste [1]. Exclusive breastfeeding is recommended up to 6 months of age with continued breastfeeding along with complementary foods up to two years of age or beyond [2].

Globally, only 37% of children younger than 6 months are exclusively breastfed [3] and increase with an annual rate of 2.4% [4]. United Nations International Children's Emergency Fund (UNICEF) and World Health Organization (WHO) are the two leading global breastfeeding initiatives to raise the rate of EBF in the first 6 months at least 50% by 2025 [3]. Early introduction of foods and liquids before 6 months of age (cessation of EBF) is common practice in all low and middle-income countries [5] and much room for improvement of EBF practice [6]. An increasing rate of breastfeeding could save the lives of more than 820,000 children under the age of 5 years [3]. There is no better substitute for breast milk, and a breastfed child is 14 times less likely to die in the first 6 months than a nonbreastfed child [7]. Promoting EBF for 6 months significantly reduces the respiratory and gastrointestinal morbidities of infants [8]. High mortality-related diarrheal disease is common in sub-Saharan countries, and early initiation of EBF reduces the risk [9]. Cessation of EBF increases the risk of pneumonia morbidity and related death [10], excessive diarrhea [11], and low growth rate [12] compared to exclusively breastfed infants. Another study conducted in Bangladesh proves that nonexclusive breastfeeding infants for the first 6 months may suffer from infectious diseases and undernutrition [13]. Children have also encountered vomiting, allergic reaction, and diarrhea immediately following the weaning process [14]. The study conducted in Northern Ethiopia showed that EBF is the strongest predictor of infant survival and which is a cost-effective, feasible, and safe strategy to reduce infant mortality [15]. EBF has also great benefits for maternal health outcomes like reducing the risk of breast and ovarian cancer, type two diabetes, postpartum depression, and have a long duration of amenorrhea or as a natural family planning method [16]. Studies showed that factors like younger mothers [17–19], low level of educational status [18–21], mothers who smoke [17, 18], low parity [19, 21–23], employed mothers [24, 25], breast problem (cracked nipple) [23], initiate of breastfeeding after one hour and had no antenatal care (ANC) visits [26], had no postnatal care (PNC) visits [26, 27], and cesarean delivery [25, 28] are some of the independent factors that push mothers to give up exclusive EBF.

Generally, increasing the practice of EBF is a fundamental driver to achieve sustainable development goals by 2030 [1]. The practice in Ethiopia is significantly lower than the global recommendation [29]. Gondar Comprehensive and Specialized Hospital is a teaching hospital that provides multiple health care services for a load of referral clients and directly comes there. However, there is limited data regarding the burden of cessation of EBF and associated factors. Therefore, this study aimed to determine the magnitude of exclusive breastfeeding cessation and to identify associated factors including knowledge-related factors that were not assessed in previous studies among mothers of infants who came for measles vaccination for their respective infant at 9 months of age.

## 2. Methods and Materials

### 2.1. Study Setting and Design

The institutional-based cross-sectional study design was conducted from February 10 to May 15, 2019, at the University of Gondar Comprehensive and Specialized Hospital, expanded program for immunization (EPI) room. This hospital is located in the Central Gondar administrative zone, Amhara National Regional State, which is far from about 750 km Northwest of Addis Ababa (the capital city of Ethiopia). According to the 2007 population and housing census report, the total population size of Gondar town was estimated to be 206,987. University of Gondar Specialized Hospital is a teaching hospital that serves more than five million people of the central Gondar zone and peoples of the neighboring zones. This hospital provided a measles vaccine for 210 infants at the age of nine months monthly, and annually 2550 infants received this immunization.

### 2.2. Study Population and Sampling Procedure

The study populations were mothers having children of 9 months age and came to vaccinate their children for measles at the University of Gondar Comprehensive Specialized Hospital during a data collection period. Any mothers other than biological mothers who brought the infants for immunization were excluded in this study. Single population proportion formula was used to calculate the sample size with the assumption of 69.63% proportion of cessation of exclusive breastfeeding before six months from the previous study in Ankesha Guagusa Woreda, AwiZone [25], 95% confidence level and 5% margin of error. After adding a 10% nonresponse rate, the final sample size was 358 mothers. Study participants were selected by using a systematic sampling method with a sampling interval (K) of two. The first mother was selected by the lottery method and continued with intervals of two until the desired sample participants achieved among mothers who came for measles vaccination at the time of data collection.

### 2.3. Variable

#### 2.3.1. Dependent Variable

Cessation of exclusive breastfeeding.

#### 2.3.2. Independent Variables

*Socio-demographic-related variables*: maternal age, religion, marital status, maternal education level, maternal occupation, and paternal education level. *Obstetric and gynecological-related factors*: parity, antenatal care (ANC) follow-up, number of visits, counseled about breastfeeding during ANC, place of delivery, mode of delivery, number of baby(ies), infant(s) sex, wanted baby(ies), early initiation of breastfeeding in 1^st^ one hour, breast problems after the most recent birth (cracked nipple, engorgement, abscess, or mastitis after birth), postnatal care (PNC) follow-up, and advised about exclusive breastfeeding during PNC follow-up. *Knowledge of mothers regarding composition and benefits of breast milk-related variables*: breast milk gives immunity to the baby, breast gives enough water for the baby, breast milk helps brain development of the baby, breastfed babies gain weight, breastfeeding is the most effective way to protect baby from diarrheal diseases, breastfeeding might be protecting mothers from breast cancer, exclusive breastfeeding helps the mother on not getting pregnant too early, breastfeeding affects mothers health badly, breastfeeding helps to build up a good bond between mother and baby, and formula milk has better benefits than breast milk. P*ersonal and breastfeeding practice-related factors*: partner support for the mother, family member influence to give feeds other than breast milk, and prior experience of exclusive breastfeeding.

### 2.4. Measurement

The data was collected using a structured questionnaire that was adapted from similar published studies with minor modifications. Initially, questionnaires are prepared by the English language, then translated to the local language (Amharic), and finally back to the English version to check its consistency. Three midwives who had a BSc degree were involved in data collection. All data collectors were oriented and well-informed about the data collection process. A pretest was done in 5% (18 mothers) of the total sample size out of the selected study population (at Addis Zemen primary hospital) to ensure the quality, clarity, understandability, and completeness of the questioner. Based on pretest input, a modification was employed on logical sequence and clarity of the questionnaire.

To gather information on cessation of EBF (fully formula feeding or partial breastfeeding) for the first 6 months was assessed by using the dietary recall method. Study participants were asked by “Did you give any solid or liquid other than breastmilk, vitamin, syrup and/or medication to the child during the first six months of age?” The mothers who had been provided considered as “yes” or ceased EBF and coded as “1” in regression analysis and those who had not considered as “No” and coded “0.”

Finally, the data were coded, entered, and analyzed by statistical package for social science (SPSS) software version 20.0. The result was presented by frequency and percent. Bivariate and multivariate logistic regressions were carried out to determine independent variables associated with cessation of exclusive breastfeeding. All variables with a *P* value of less than 0.2 in the bivariate logistic regression model were included in multivariate logistic regression, and variables with *P* value less than 0.05 were considered as significantly associated independent factors.

### 2.5. Operational Definition

*Cessation of exclusive breastfeeding*: The report that the mother introduces any liquid (like formula milk, water, or/and juice) or solids (like infant cereal) except drops or syrups consisting of vitamins, mineral supplements, or medications for her infant aged less than six months [25].

*Good and poor knowledge*: Mothers who gave a minimum of 5 out of 10 or 50% correct answer of knowledge-related questions are considered as mothers that had good knowledge, if not, considered as having poor knowledge [30].

### 2.6. Ethical Considerations

Ethical clearance was obtained from the ethical review board committee of the University of Gondar, College of Medicine and Health Science and School of Midwifery. A supportive letter was given for the University of Gondar referral hospital immunization room staff to get permission. After the purpose and objective of the study informed, written consent was obtained from each study participant. Participation was entirely voluntary and mothers were informed about the right to withdraw from the study at any time of the interview. The participants were also assured that the data was confidential.

## 3. Results

### 3.1. Socio-Demographic-Related Characteristics of Respondents

In this study, a total of 344 mothers have participated with a response rate of 99.04%. The mean (±SD) age of the mothers was 26.41 (±4.312) years with a minimum and maximum of 18 and 40 years, respectively. Almost all (99.4%) were married and majorities (87.2%) were orthodox religious followers. Nearly, three fourth (74.1%) of the respondents were with the age range of 20–29 years. More than half (53.5%) of the mothers were housewives by occupation, and 147 (42.7%) participants completed college and above by their educational background. Three hundred eighteen (92.4%) came from urban areas. More than half (54.4%) and 161 (46.8%) mothers' partners have completed college education and had paid jobs, respectively (Table 1).

### 3.2. Obstetrics and Gynecology-Related Factors

Half (50.9%) of the respondents gave birth to a viable fetus once (primi-para). Almost all (97.4%) mothers attended ANC follow-up. From those respondents who had ANC follow-up, 292 (87.2%) and 149 (44.5%) of them had ≥4 visits and received counseling on infant feeding during the follow-up, respectively. Only 14 (4.1%) mothers gave birth at home. More than half (57.6%) of the mothers gave birth via spontaneous vaginal delivery, and 96.2% of babies were wanted. One third (33.4%) of the mothers had no PNC follow-up (Table 2).

### 3.3. Knowledge of Mothers on the Composition and Benefits of Breast Milk

Nearly one fifth, or 17.2% and 23.3%, of mothers did not know that breast milk has antibodies and breast milk is the most effective way to prevent babies from diarrheal disease, respectively. One third (32.6%) of the respondents believed that breast milk had no enough water for the baby. Half (51.7%) of the respondents knew breastfeeding protects mothers from acquiring breast cancer, and 119 (34.6%) respondents believed that formula milk has better benefits than breast milk. Generally from the total participants, 284 (82.6%) of those respondents had good knowledge or gave the correct answer of ≥5 out of 10 knowledge-related questions about the composition and benefits of breast milk assessment questions (Table 3).

### 3.4. Personal and Breastfeeding Practice-Related Factors

More than three fourth of the participants (90.1%) were supported by their partner at home and 26 (7.6%) participants were influenced by their family members to give complementary feeding than EBF up to 6 months. Thirty-seven (10.8%) had prior experience of exclusive breastfeeding. Primi-para mothers had no prior experience. From a total of 74 (21.7%) mothers who had cessation-exclusive breastfeeding, nearly half (44.6%) mothers discontinued within 1–3 months of infant age (Table 4).

### 3.5. Magnitude and Reasons for Cessation of EBF

**Figure 1:**
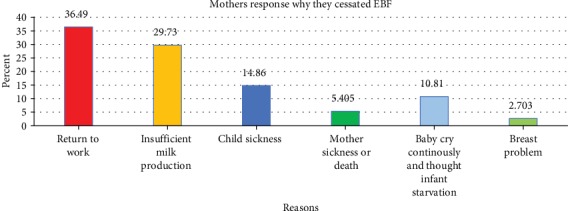
Reasons why mothers started any kind of fluid or/and solid diet for their infant before 6 months of age at University of Gondar Comprehensive and Specialized Hospital, Northwest Ethiopia, 2019 (*n* = 74).

Seventy-four (21.5%) mothers were discontinued EBF before 6 months. The mean month to discontinue EBF was 2.49 with a standard deviation of ±1.73 months. From the total of 74 mothers who had cessation EBF, 27 (36.5%) mothers replied “return to work” as a reason (Figure 1).

### 3.6. Factors Associated with Cessation of Exclusive Breastfeeding

In bivariate logistic regression model ten variables like age ≤ 19 years, civil servant by occupation, illiterate husbands (not writing or reading), being primi-para, gave birth assisted by instruments, gave birth at health center, had no PNC follow up, did not initiate breastfeeding early (within 1 hour), breast-related problem after birth, and had poor knowledge on benefits and compositions of breast milk were variables associated with cessation of EBF with *P* value <0.2. After adjusting possible confounders, age ≤ 19 years, a civil servant by occupation, illiterate (not writing or reading), being primi-para, had no PNC follow-up, and had poor knowledge on benefits and compositions of breast milk were found to have a statistically significant association with the cessation of EBF (Table 5).

## 4. Discussion

### 4.1. Cessation of Exclusive Breastfeeding Practice

The magnitude of cessation of EBF was found to be 21.5% (*n* = 74) with 95% CI (17.24-25.76). The magnitude in this study was in line with the study conducted in India (22.85%) [28] and slightly lower than Southeast Ethiopia (28.7%) [24], Tigray (29.8%) [27], and Canada (28.4%) [31]. This slight difference could be t these studies were community-based and conducted before four years of this study and the study conducted in Canada was longitudinal. However, the magnitude of cessation of EBF was lower than what had been reported in Australia (38%) [17], Brazil (39.7%) [23], and Sri Lanka (49.2%) [30]. The disparities might be due to study design, level of understanding regarding the studies and telling reliable data, and have good additional feeding style since the above countries are in the high and middle-income economic category.

The study conducted in Malaysia (45.6%) [32], Taiwan (70.7%) [21], Democratic Republic of Congo (97.2%) [33], Sorro district, South Ethiopia (49.4%) [26], and AnkeshaGuagusa Woreda (69.63%) [25] were also having a high prevalence rate of cessation of EBF compared to this study. The dissimilarities could be due to the studies conducted other than Taiwan were community-based which is more representative and used mixed (both qualitative and quantitative) method, socio-cultural difference, and study setting. The study in Taiwan had used a large sample size, and a wide range of study periods could be the reason for the difference.

### 4.2. Determinant Factors with the Cessation of Exclusive Breastfeeding

Independent factors that were statistically associated with cessation of EBF included parity, maternal occupation, maternal age, paternal level education, PNC, and knowledge on benefits and composition of EBF.

Younger women (age ≤ 19 years) were 5.53 times at higher odds to discontinue EBF before 6 months of infant age than mothers age ≥30 years. This is consistent with the finding of Canada [18, 31], Australia [17], Bangladesh [19], North Ethiopia [27], and Northwest Ethiopia [25]. This could be explained as younger women possibly have a low level of understanding about EBF and the consequence of discontinuing breastfeeding on their infants. Additionally, most mothers believe that breastfeeding might hurt their breast shape (need to have the same shape as a nonpregnant state).

Mothers who had been civil servants (employed) by occupation were 4.73 times more likely to cessation EBF compared to those housewife mothers. This is supported by the study conducted in Canada [18], Sri Lanka [30], Bangladesh [19], Taiwan [21], Goba district, Southeast Ethiopia [24], and Northwest Ethiopia [25]. This could be because civil servant mothers may return to the workplace before 6 months of infant age and perceive their infant starved. Then, they will have a high likelihood of start other fluids or diets. Mothers who had illiterate (no educational background) partner was 3.76 times more likely to stop EBF compared to those partners who had an educational status of college and above. No studies support this finding. But, the study conducted in Northwest Ethiopia [25] revealed that partner educational status of college and above were 70% more likely to discontinue compared to none educated. The reason could be the study conducted in Northwest Ethiopia was community-based and in the rural area. Therefore, cutural and traditional thoughts may affect more despite husbands' high level of educational background. Husbands who had low educational background obviously will never involve in infant care and have poor knowledge of recommended infant feeding in the first 6 months.

Like many other previous studies, the odds of cessation of EBF among primi-para mothers were 2.42 times higher than their counterparts. The finding was similar to the study conducted in England [22], Bangladesh [19], Taiwan [21], and northwest Ethiopia [25]. The reason explained that primi-para mothers had no prior experience of breastfeeding and may not realize how much breast milk for the first 6 months is best of breastfeeding. Women who had no postnatal care (PNC) visits were 2.62 times more likely to discontinue EBF before 6 months compared to women who had PNC visits. This is in agreement with the study conducted in South Ethiopia [26], North Ethiopia [27], and Northwest Ethiopia [25]. The explanation could be that mothers who had PNC follow-up received information from health care providers on duration, frequency, compositions, and benefits of EBF.

Mothers who had no good knowledge (not knowledgeable) were 3.15 times more likely to cessation EBF compared to their counterparts. The study conducted in the Democratic Republic Congo [33] agreed with this finding. This might be explained because mothers who had good knowledge about the compositions and benefits of breast milk would have good practice rate of EBF.

### 4.3. Limitation

This study has some limitations. Cessation of EBF is better to be assessed by asking about "the last 24 hour dietary practice preceding the interview" or through longitudinal follow-up approach. However, due to time and financial constraints, cessation of EBF was assessed through client self-reporting question retrospectively about the entire 6 months of age feeding practice which was challenging to recall cessation of exclusive breastfeeding or not. To minimize this effect, we tried to encourage mothers correlated to different events like “baptism” or “Christina” in Amharic for those orthodox Christian followers to remember when they initiated other than breastmilk in the month instead of in date to remember easily. Since the study design was cross-sectional, it is not possible to establish a true cause and effect relationship. Participants were selected among mothers who came for measles immunization of their infants at 9 months of age, which might not be representative of women who did not come for immunization was the last but not the least limitation of this study.

### 4.4. Conclusion

The magnitude of the cessation of exclusive breastfeeding was high. Factors determining EBF cessation were maternal age, civil servant, parity, postnatal visits, and breastfeeding knowledge, as well as spouse literacy. Critical to minimizing EBF cessation are increasing awareness on breastfeeding, improving postnatal follow-ups (up to community level), extending maternal leave (e.g., for up to 6 months), and supporting breastfeeding at the workplace.

## Figures and Tables

**Table 1:** Socio-demographic characteristics of the respondents at University of Gondar Comprehensive and Specialized Hospital, Northwest Ethiopia, 2019 (*n* = 344).

Variable	Category	Frequency	Percent (%)
Maternal age in years	<20	11	3.2
	20–29	255	74.1
	≥30	78	22.7

Religion	Orthodox	300	87.2
	Muslim	37	10.8
	Protestant	7	2.0

Educational status	No education	32	9.3
	Primary	70	20.3
	Secondary	95	27.6
	College and above	147	42.7

Marital status	Married	342	99.4
	Widowed	1	0.3
	Divorced	1	0.3

Maternal occupation	Housewife	184	53.5
	Merchant	32	9.3
	Civil servant	109	31.7
	Private sector	19	5.5

Occupation of the partner	Farmer	31	9.0
	Paid job	161	46.8
	Student	6	1.7
	Merchant	99	28.8
	Others	47	13.7

Educational status of the partner	No education	22	6.4
	Primary	50	14.5
	Secondary	85	24.7
	College and above	187	54.4

Place of residence	Rural	26	7.6
	Urban	318	92.4

**Table 2:** Obstetrical and gynecological-related factors of respondents at University of Gondar Comprehensive and Specialized Hospital, Northwest Ethiopia, 2019 (*n* = 344).

Variable	Category	Frequency	Percent (%)
Parity	*Primi-para*	175	50.9
	*Multi-para*	169	49.1

ANC follow-up	Yes	335	97.4
	No	9	2.6

Number of ANC visits (*n* = 335)	<4	43	12.8
	≥4	292	87.2

Counsel on infant feeding during ANC follow-up (*n* = 335)	Yes	149	44.5
	No	186	55.5

Place of birth	Home	14	4.1
	Health center	21	6.1
	Hospital	309	89.8

Mode of delivery	SVD	198	57.6
	Instrumental	72	20.9
	C/S	74	21.5

Number of babies delivered	Singleton	337	98.0
	Multiple	7	2.0

Sex of the baby	Male	171	49.7
	Female	167	48.6
	Male and female	6	1.7

Baby wanted	Yes	331	96.2
	No	13	3.8

Breastfeeding within one hour of birth	Yes	285	82.8
	No	59	17.2

Breast problems (cracked nipple, engorgement, abscess, or mastitis) after delivery	Yes	48	14.0
	No	296	86.0

PNC follow-up	Yes	229	66.6
	No	115	33.4

Counsel about EBF during PNC follow-up (*n* = 229)	Yes	130	56.8
	No	99	43.2

**Table 3:** Knowledge of mothers on compositions and benefits of breast milk at University of Gondar Comprehensive and Specialized Hospital, Northwest Ethiopia, 2019 (*n* = 344).

Variable	Category	Frequency	Percent (%)
Breast milk gives immunity to the body	Yes	285	82.8
	No	59	17.2

Breast milk gives enough water to the baby	Yes	232	67.4
	No	112	32.6

Breast milk helps brain development of the baby	Yes	289	84.0
	No	55	16.0

Breastfeed baby gains weight	Yes	292	84.9
	No	51	14.8
	I do not know	1	0.3

Breastfeeding is the most effective way to prevent baby from diarrheal diseases	Yes	264	76.7
	No	80	23.3

Breastfeed might protect mother from breast cancer	Yes	178	51.7
	No	145	42.2
	I do not know	21	6.1

EBF helps the mother not to get pregnant early	True	214	62.2
	False	130	37.8

Breastfeeding helps to build up bonding between mother and baby	True	306	89.0
	False	38	11.0

Formula milk has better benefits than breast milk	True	119	34.6
	False	225	65.4

Breastfeeding affects maternal health badly	True	102	29.7
	False	242	70.3

**Table 4:** Personal and breastfeeding practice-related factors of respondents at the University of Gondar Comprehensive and Specialized Hospital, northwest Ethiopia, 2019 (*n* = 344).

Variable	Category	Frequency	Percent (%)
Does your partner support you at home?	Yes	310	90.1
	No	34	9.9

Family members influenced to give complementary feeding than EBF up to 6 months	No	318	92.4
	Yes	26	7.6

Prior exclusive breastfeeding experience (Para 1 response considered as “no”)	No	307	89.2
	Yes	37	10.8

The month when the mother initiated a fluid/solid diet before 6 months (*n* = 74)	Within 1 month	13	17.6
	1-3 months	33	44.6
	≥4 months	28	37.8

**Table 5:** Bivariate and multivariable logistic regression of factors associated with cessation of exclusive breastfeeding at University of Gondar Comprehensive and Specialized Hospital, Northwest Ethiopia, 2019 (*n* = 344).

Variable	Category	Cessation of EBF		COR (95% CI)	AOR (95% CI)
		No	Yes		
Age in years	≤19	5(45.5%)	6 (54.5%)	4.65 [1.26-17.2]	**5.53 [1.07-28.57]** ^∗^
	20-29	203 (79.6%)	52 (20.4%)	—	—
	≥30	62 (79.5%)	16 (20.5%)	1.00	1.00

Maternal occupation	Housewife	155 (84.2%)	29 (15.8%)	1.00	1.00
	Merchant	27 (84.4%)	5 (15.6%)	—	—
	Civil servant	74 (67.9%)	35 (32.1%)	2.52 [1.44-4.45]	**4.73 [2.20-10.19]** ^∗^
	Private sector	14 (73.7%)	5 (26.3%)	—	—

Paternal level of education	No education	14 (63.6%)	8 (36.4%)	2.24 [0.88-5.73]	**3.76 [1.13-12.49]** ^∗^
	Primary	44 (88.0%)	6 (12.0%)	0.53 [0.21-1.34]	0.76 [0.25-2.38]
	Secondary	63 (74.1)	22 (25.9%)	—	—
	College and above	149 (79.7)	38 (20.3%)	1.00	—

Parity	Primi-para	125 (71.4%)	50 (28.6%)	2.41 [1.41-4.16]	**2.42 [1.22-4.79]** ^∗^
	Multipara	145 (85.8%)	24 (14.2%)	1.00	—

Place of birth	Home	10 (71.4%)	4 (28.6%)	—	—
	Health center	14 (66.7%)	7 (33.3%)	1.95 [0.76-5.04]	1.55 [0.51-4.75]
	Hospital	246 (79.%)	63 (20.4%)	1.00	—

Mode of delivery	SVD	165 (83.3%)	33 (16.7%)	1.00	—
	IAVD	48 (66.7%)	24 (33.3%)	2.50 [1.35-4.63]	2.01 [0.97-4.19]
	C/S	57 (77.0)	17 (23.0%)	—	—

Early initiation of BF	Yes	23 (81.1%)	54 (18.9%)	1.00	—
	No	39 (66.1%)	20 (33.9%)	2.19 [1.19-4.06]	1.62 [0.72-3.63]

Breast related problem	Yes	32 (66.7%)	16 (33.3%)	2.05 [1.06-3.99]	2.17 [0.99-4.74]
	No	238 (80.4%)	58 (19.6%)	1.00	

PNC	Yes	192 (83.8)	37 (16.2%)	2 1.00	—
	No	78 (67.8%)	37 (32.2%)	2.46 [1.45-4.17]	**2.62 [1.44-4.80]** ^∗^

BF affect maternal health badly	False	196 (72.6%)	46 (62.2%)	1.00	—
	True	74 (27.4%)	28 (37.8%)	1.61 [0.93-2.77]	1.2 [0.65-2.20]

FM better benefits than BM	False	183 (67.8%)	42 (56.8%)	**1.00**	**—**
	True	87 (32.2%)	32 (43.2%)	1.60 [0.95-2.71]	1.31 [0.75-2.28]

BF prevents from diarrheal diseases	True	215 (79.6%)	49 (66.2%)	1.00	**—**
	False	55 (20.4%)	25 (33.8%)	1.99 [1.13-3.51]	1.63 [0.89-2.97]

Breast milk gives enough water	True	192 (71.1%)	40 (54.1%)	1.00	**—**
	False	78 (28.9%)	34 (45.9%)	2.09 [1.26-3.55]	**2.19 [1.26-3.74]** ^∗^

Knowledge of benefit and compositions of BF	Good	233 (82.0%)	51 (18.0%)	1.00	—
	Poor	37 (61.7%)	23 (38.3%)	2.84 [1.55-5.19]	**3.15 [1.56-6.35]** ^∗^

^∗^Significantly associated variables with *P* value < 0.05 and 1.00 represent reference variable. FM: formula feeding; BF: breastfeeding; PNC: postnatal care; SVD: spontaneous vaginal delivery; IAVD: instrumental assisted vaginal delivery; C/S: cesarean section; EBF: exclusive breastfeeding.

## Data Availability

The data used to support the findings of this study are available from the corresponding author upon request.

## Abbreviations

AOR: Adjusted odds ratio
ANC: Antenatal care
COR: Crude odds ratio
CI: Confidence interval
EBF: Exclusive breastfeeding
PNC: Postnatal care.
